# A Novel Mutation in a Kazakh Family with X-Linked Alport Syndrome

**DOI:** 10.1371/journal.pone.0132010

**Published:** 2015-07-13

**Authors:** Barshagul T. Baikara, Elena V. Zholdybayeva, Saule E. Rakhimova, Nazym B. Nigmatullina, Kuvat T. Momynaliev, Yerlan M. Ramanculov

**Affiliations:** 1 National Center for Biotechnology, Astana, Kazakhstan; 2 Center for Sciences, Nazarbayev University, Astana, Kazakhstan; 3 National Research Center for Maternal and Child Health, Astana, Kazakhstan; 4 School of Science and Technology, Nazarbayev University, Astana, Kazakhstan; Estonian Biocentre, ESTONIA

## Abstract

Alport syndrome is a genetic condition that results in hematuria, progressive renal impairment, hearing loss, and occasionally lenticonus and retinopathy. Approximately 80% of Alport syndrome cases are caused by X-linked mutations in the *COL4A5* gene encoding type IV collagen. The objective of this study was to define the SNP profiles for *COL4A5* in patients with hereditary nephritis and hematuria. For this, we examined four subjects from one Kazakh family clinically affected with X-linked Alport syndrome due to *COL4A5* gene mutations. All 51 exons of the *COL4A5* gene were screened by linkage analysis and direct DNA sequencing, resulting in the identification of a novel mutation (G641E) in exon 25. The mutation was found only in two affected family individuals but was not present in healthy family members or 200 unrelated healthy controls. This result demonstrates that this novel mutation is pathogenic and has meaningful implications for the diagnosis of patients with Alport syndrome.

## Introduction

In 1927, Alport reported that deafness was a feature of a previously described familial nephropathy that caused uremia specifically in males [1]. Subsequent studies found that this renal dysfunction results from splitting of the glomerular basement membrane (GBM), hematuria, interstitial nephritis, and progressive kidney failure [2]; however, the genetic basis for Alport syndrome was unknown until mutations in the *COL4A3*, *COL4A4*, and *COL4A5* collagen genes were discovered in clinically affected families [3–6]. Collagen proteins consist of an N-terminal 7S domain and a C-terminal non-collagenous (NC1) domain linked by a large collagenous domain that contains a characteristic Gly-X-Y repeat common to all collagens [7].

Alport syndrome is an inherited disease characterized by hematuria and/or proteinuria, and occasionally sensorineural hearing loss and eye lesions, which progresses to chronic renal failure [8]. Alport syndrome is a genetically heterogeneous disease; however, the pathogenesis of X-linked Alport syndrome is attributed to mutations in one of the three genes encoding type IV collagen α-chain isoforms (α3, α4, and α5). These genes are necessary for proper GBM development, which plays a crucial role in the purification of blood plasma in the kidney [6].

Approximately 80% of X-linked Alport syndrome cases result from mutations in the collagen α5 (IV) chain gene (*COL4A5*) on chromosome Xq24–48 [5, 9–11]. These patients develop end-stage renal disease (ESRD), with an age-associated risk of onset: 50% by age 25, 90% by age 40, and nearly 100% by age 60 [4]. Autosomal recessive and dominant forms also exist due to mutations in *COL4A3* or *COL4A4* that present in 15% and 5% of cases, respectively [12].

X-linked Alport syndrome is one of the most severe forms of inherited glomerulopathy and is characterized by the alternating thinning and thickening of the GBM, as well as splitting and lamellation of the lamina densa. At a young age, affected hemizygous males and heterozygous female carriers may present initially with a uniform thinning of the GBM, which progresses rapidly in affected males to more severe thickened and lamellated structure. This is traditionally accompanied by the onset of proteinuria, hypertension, and renal failure, subsequently leading to ESRD sometime between adolescence and 30 years of age. In addition, sensorineural hearing loss and ocular abnormalities are present in approximately 82% and 44% of Alport syndrome patients, respectively [4].

In this study, we have analyzed *COL4A5* mutations and disease characteristics in a Kazakh family with X-linked Alport syndrome. Clinical examinations revealed that all affected family members displayed renal dysfunction and hearing abnormalities, while ocular abnormalities were only observed in the proband. Moreover, we identified a novel pathogenic mutation determining the amino acid change of glycine to glytamic acid at position 641 of the protein codified by the COL4A5 gene, which may be clinically significant. Altogether, our data broadens the genotypic spectrum of *COL4A5* mutations associated with mild Alport syndrome.

## Materials and Methods

### Ethical Statement

This study was approved by the Local Ethics Committee of the National Center for Biotechnology, (No. 2, 12.03.2012) and was conducted in accordance with humane and ethical research principles. All study participants or their parents completed a questionnaire and signed informed consent. In addition, the family members with Alport syndrome have provided all necessary information pertaining to their family history.

### Study subjects

The Kazakh family with multiple individuals affected with Alport syndrome is from Aktau, Kazakhstan. Clinical diagnosis was made following a comprehensive physical exam by a consulting ophthalmologist and an ENT surgeon. Ocular examination included testing for visual acuity, anterior lenticonus, cataracts, and optic disc or retinal abnormalities. Sensorineural hearing loss was assessed using standard audiometry tests.

The affected familial pedigree consisted of four individuals across four generations. The proband is a 6-year-old girl with chronic glomerulonephritis and a familial history of hematuria. She was full-term at birth (birth weight, 3500 g), and hematuria first appeared at 1 year of age with traces of protein and red blood cells (erythrocytes) in urine. She was observed and diagnosed in June 2013 at the National Research Center for Maternal and Child Health in Astana. Urine analysis showed signs of proteinuria (0.11 g/day) and gross hematuria. Clinical laboratory analysis revealed the following: hemoglobin (Hg) concentration (129 g/L), erythrocyte sedimentation rate (ESR; 4 mm/h), creatinine (31 mmol/L), glomerular filtration rate (143 mL/min), normal complete blood counts (leukocytes, 2–3 × 10^9^/L; erythrocytes, 10–12 × 10^9^/L), and anti-neutrophilic cytoplasmic antibody (ANCA) is negative. Renal ultrasonography showed diffuse parenchymal changes in both kidneys without disturbance hemodynamics. External symptoms such as edema or hypertension were not observed (blood pressure 91/60).

Upon examination for this study, she was also diagnosed with acute nasopharyngitis and retinal vessel angiopathy. Audiogram confirmed normal hearing.

This patient is currently under observation by physicians and on nephroprotective therapy. In 2014, she appeared healthy with a normal weight and height (weight, 21.5 kg; height, 117 cm). Her blood analysis showed an elevated Hg concentration (108 g/L) and increased ESR (25 mm/h), with a normal blood count (leukocytes, 6.73 × 10^9^/L; erythrocytes-4.09 × 10^9^/L). Analysis of renal function showed a creatinine blood concentration of 29 mmol/L and a glomerular filtration rate at 161 mL/min. Urinalysis revealed a protein excretion of 0.05 g/day. At the time of this study (one year after the primary assessment), renal ultrasonography failed to identify any structural changes and hemodynamic disturbances; however, the patient did display an increased glomerular filtration rate.

**Fig 1:**
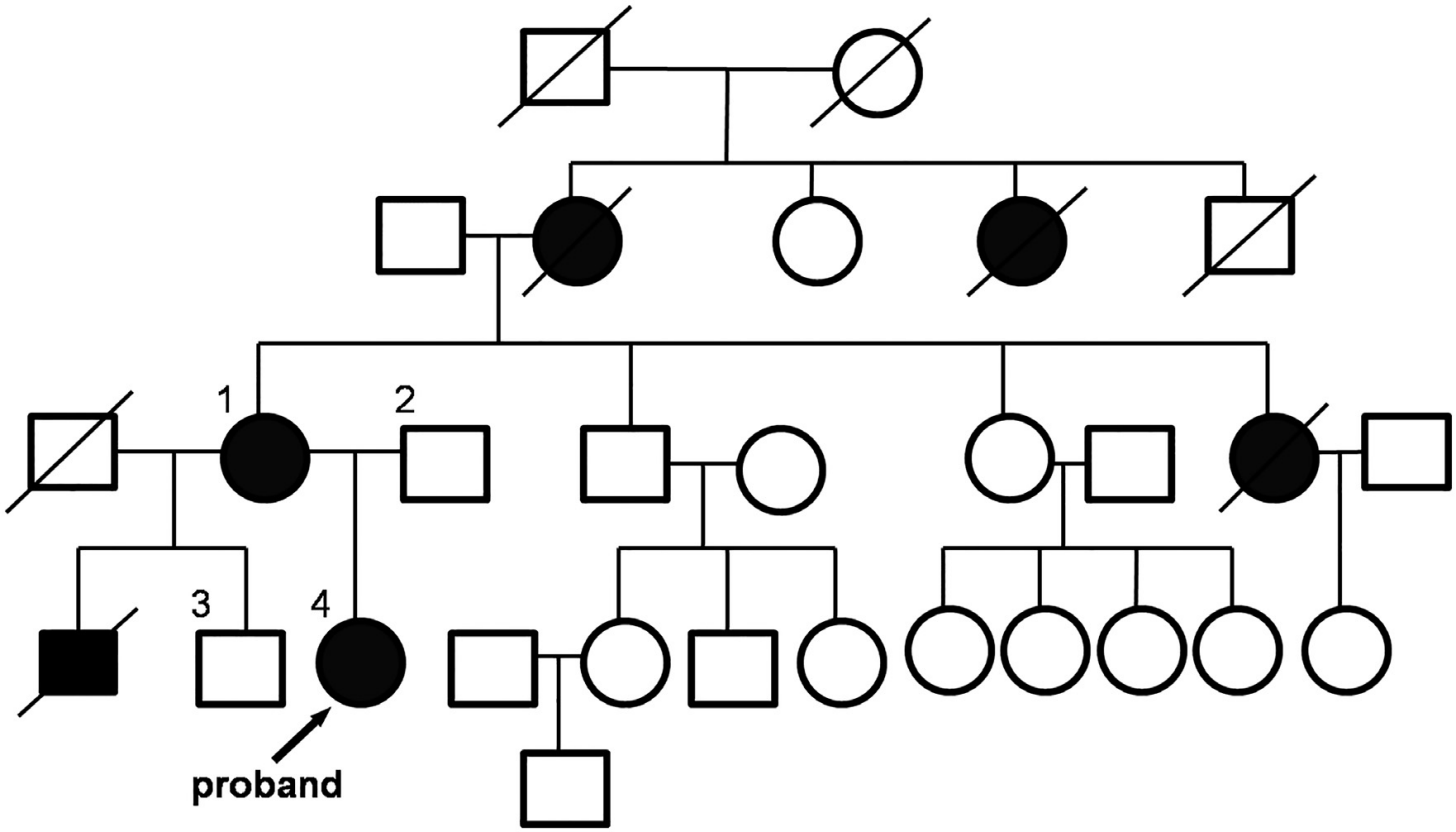
Pedigree of a Kazakh Family with X-Linked Alport Syndrome. Affected individuals with kidney disease are shown with blackened squares (males) and circles (females). Normal individuals are shown with empty squares (males) and circles (females). Crossed squares or circles denote deceased individuals.

During the study of family history, we found that the proband’s older brother died from glomerulonephritis at age 12. Her father (49-years-old) and 24-year-old older brother are healthy. The proband’s mother (44-years-old) was diagnosed with pyelonephritis and hearing loss. The mother has a healthy brother (47-years-old) and a sister (42-years-old), but had a younger sister that died of glomerulonephritis and kidney disease at age 29. In addition, the proband’s maternal grandmother was affected by arterial hypertension, chronic renal failure, and died at the age of 31. Signs of arterial hypertension were also identified in her maternal great-aunt. The family’s pedigree is shown in Fig 1. Based on family history, we have identified the disease and inheritance pattern as X-linked Alport syndrome resulting from mutations in *COL4A5*.

Peripheral blood samples were also collected from 200 unrelated, ethnically normal controls. All participants underwent clinical examination by an ophthalmologist and otolaryngologist. Volunteers with vision and/or hearing abnormalities, hypertension, or blood concentrations of creatinine or urea outside of normal levels were excluded from the study.

### Mutational analysis

Genomic DNA was extracted from peripheral whole blood samples by using Wizard Genomic DNA Purification Kit (Promega, Madison, WI). All exons and intron-exon junctions in *COL4A5* (NM_000495) were amplified by PCR. Mutational analysis was performed by direct DNA sequencing using protocols recommended by the reference laboratory at the University of Utah. PCR products were run on 1.2% agarose gel and sequenced with both forward and reverse primers. DNA sequencing was carried out using the BigDye Terminator Cycle Sequencing v3.1 kit (ABI, Foster City, CA). Automated sequencing was performed on 3730 XL Genetic Analyzer (Applied Biosystems, Life Technologies, CA) in order to determine whether the mutation co-segregated with the disease in the family, and if was present in any of the 200 normal controls.

## Results

**Fig 2:**
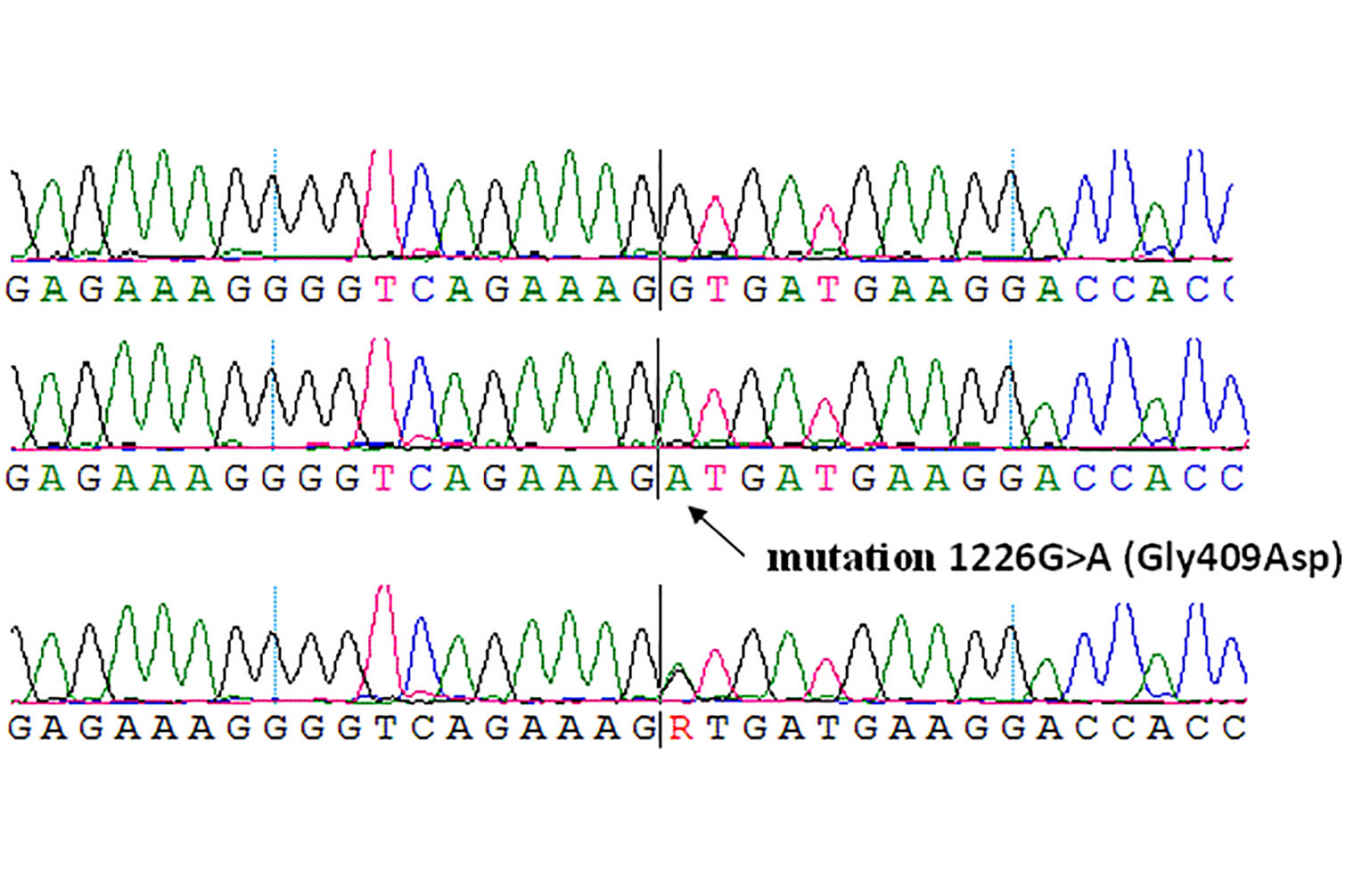
Identification of a Novel *COL4A5* Mutation in a Family with X-Linked Alport Syndrome. A. Exon 25 sequencing from a normal male individual. B. Sequencing from a heterozygous female. C. G641E is a mutation that results in 48 abnormal amino acid residues in the COL4A5 protein.

In this study, we sequenced 51 exons of the *COL4A5* gene in four family members. From this, we identified a nucleotide change 1922 G>A, causing amino acid substitutions of glycine to glytamic acid at position 641, in exon 25 of the *COL4A5* gene. The glycine 641 may be critical for the proper conformation of the Collagen α5 (IV) protein. As a result, it was found out that the proband and her mother are heterozygous for the G641E mutation (Fig 2), both of which exhibit phenotypic characteristics including sensorineural hearing loss and eye disease (retinal vessel angiopathy). This mutation was not detected in the proband’s father or brother.

Direct DNA sequence analysis of family members showed that the mutation co-segregated with the disease, and was not present in unaffected family members or the 200 normal control individuals. This result demonstrates that this novel mutation of *COL4A5* is not a SNP, but a pathogenic mutation causing Alport syndrome.

## Discussion

In this study, we investigated a Kazakh family with Alport syndrome. Through linkage analysis and direct DNA sequencing, we identified a novel mutation G641E in the *COL4A5* gene in two family members. The glycine 641 residue may be critical for the proper conformation of the collagen α5(IV) protein. Most reported *COL4A5* mutations are missense mutations located primarily in the collagenous portion of the α5-collagen chains that incorporate conserved glycine residues important for the steric arrangement of the collagen chain [13]. Wang et al. previously studied the effect of glycine substitutions (G1015V and G1030S) on the collagen α5(IV) chain structure and structure-phenotype correlations in Alport syndrome. They concluded that the changes in the secondary protein structure might explain the phenotypic diversities of X-linked Alport syndrome [14]. In addition, glycine substitutions in the collagenous domain of the α5 (IV)-chain will result in an adult form of Alport syndrome with a mean ESRD onset age of 35.8 years [14,15]. However, the proband of our study exhibited renal impairment during the first year of life, and her brother died from glomerulonephritis at age 12.

Since the proband’s brother, maternal grandmother, and younger sister all succumbed to kidney disease, we did not have a chance to analyze their DNA. Nevertheless, sequence analysis showed that the mutation was not present in healthy of family members or the 200 healthy control individuals.

The vast majority of affected children with X-linked Alport syndrome have parent with hematuria; however, normal urinalyses in the parents of a child with hematuria cannot exclude a diagnosis of X-linked Alport syndrome [16].

After a clinical and molecular investigation of hundreds of X-linked Alport cases over the past two decades, a significant amount of data has been accumulated regarding the importance of correlating patient genotype to the observed phenotype. Two forms of X-linked Alport syndrome are usually recognized based on the age at ESRD onset and the rate of clinical progression: the classical, well-recognized "juvenile" type and the "adult" type [8]. In the former, ESRD ensues rapidly in men in their twenties, whereas ESRD presents in a delayed fashion in the later [17].

Given that the proband’s brother died of ESRD, we can assume that this mutation in hemizygous males leads to serious renal complications, ESRD, and death, indicative of a "juvenile" type.

In conclusion, the identification of new mutations and their associated phenotypes are very important to predict disease prognosis, clarify their clinical importance, and to provide better genetic counseling for affected families.
